# Integrative conservative rehabilitation for adolescent idiopathic scoliosis: a randomized controlled trial of traditional Chinese manual therapy combined with Schroth training

**DOI:** 10.3389/fped.2026.1868538

**Published:** 2026-07-30

**Authors:** Junhai Li, Yu Zhang, Kewei Ying, Youxin Lv, Jiajia Zhou

**Affiliations:** 1 Department of Child Healthcare, Wenzhou People’s Hospital, Zhejiang, China; 2 Department of Pediatrics, Wenzhou People’s Hospital, Zhejiang, China

**Keywords:** adolescent idiopathic scoliosis, cobb angle, rehabilitation, schroth exercises, traditional Chinese manual therapy

## Abstract

**Background:**

Adolescent idiopathic scoliosis (AIS) is a three-dimensional spinal deformity that can lead to postural imbalance, functional limitations, and reduced quality of life. While Schroth exercises are established as an effective conservative intervention, the potential benefits of combining Traditional Chinese manual therapy (TCM) with Schroth exercises remain underexplored.

**Objective:**

To evaluate the efficacy and safety of TCM combined with Schroth exercises compared with Schroth exercises alone in adolescents with AIS. The primary objective was to determine whether the combined intervention results in greater improvement in Cobb angle over 12 weeks.

**Methods:**

*n* this single-center, randomized, parallel-group controlled trial, 86 adolescents with AIS (Cobb angle 10°–45°, age 8–18 years) were randomized 1:1 to receive either Traditional Chinese manual therapy plus Schroth exercises (*n* = 43) or Schroth exercises alone (*n* = 43). Participants receiving brace treatment were excluded, and no bracing was permitted during the study. The intervention lasted 12 weeks, including a 2-week supervised phase followed by a 10-week home program. The primary outcome was change in Cobb angle from baseline to week 12. Secondary outcomes included angle of trunk rotation (ATR), paraspinal thermal asymmetry (ΔT), and health-related quality of life (SRS-22r). A follow-up assessment at 2 weeks was included to capture early treatment response, but the study was not powered to evaluate trajectory or time-to-maximum correction. Analyses were performed using ANCOVA and linear mixed-effects models under an intention-to-treat framework.

**Results:**

The combined intervention group showed greater improvement in Cobb angle compared with the control group (mean difference in change: −2.5°, *p* < 0.001). Significant between-group differences were also observed in ATR reduction, ΔT change, and SRS-22r improvement. An early improvement pattern was observed, with a substantial proportion of total change occurring within the first 2 weeks, suggesting rapid initial adaptation rather than confirmed sustained structural correction. Both interventions were well tolerated, and no serious adverse events were reported.

**Conclusion:**

Traditional Chinese manual therapy combined with Schroth exercises was associated with short-term improvements in spinal alignment and quality of life compared with Schroth exercises alone in adolescents with AIS. However, these findings reflect early short-term treatment effects and should not be interpreted as evidence of long-term stability or curve progression control. Further multicenter studies with longer follow-up are required to confirm durability and clinical relevance.

## 1 Introduction

Adolescent idiopathic scoliosis (AIS) is a complex three-dimensional spinal deformity that develops during periods of rapid skeletal growth, typically between 10 and 18 years of age. It is characterized by lateral curvature, vertebral rotation, and alterations in sagittal alignment, and affects approximately 2%–3% of adolescents worldwide (1, 2). Although many cases remain mild, progressive deformity can lead to trunk asymmetry, back pain, impaired pulmonary function, and reduced health-related quality of life, as well as psychological distress related to body image and self-esteem (1, 3). Therefore, early identification and effective conservative management are essential to prevent curve progression and minimize long-term morbidity.

Current management strategies for AIS are largely guided by curve severity and skeletal maturity. For mild-to-moderate curves (Cobb angle 10°–45°), conservative treatment remains the cornerstone of care, with the primary goal of preventing progression and improving functional outcomes without surgery (4). According to current clinical guidelines, bracing is the standard non-operative treatment for moderate AIS in skeletally immature patients, particularly for curves with higher progression risk, and is often used in combination with physiotherapeutic scoliosis-specific exercises (PSSE) to optimize outcomes. Surgical intervention is generally reserved for severe or progressive deformities that fail conservative management. Among conservative approaches, physiotherapeutic scoliosis-specific exercises (PSSE) have gained widespread recognition, particularly the Schroth method. This method focuses on three-dimensional auto-correction, rotational angular breathing, postural stabilization, and sensorimotor integration tailored to individual curve patterns.

Recent high-quality studies and systematic reviews have demonstrated that Schroth exercises can significantly improve Cobb angle, angle of trunk rotation (ATR), and quality of life in adolescents with AIS (4–6). For example, meta-analyses indicate that Schroth-based interventions are associated with reductions in spinal curvature and improvements in patient-reported outcomes compared with conventional or no treatment (4–6). Moreover, long-term supervised programs appear to produce more sustained benefits, suggesting a dose–response relationship between exercise adherence and clinical improvement (6). Despite these promising findings, the magnitude of structural correction achieved with exercise alone remains modest, and evidence quality is limited by heterogeneity in study design, intervention protocols, and adherence levels (4).

AIS is not only a structural deformity but also involves complex alterations in muscle activity, soft tissue properties, and biomechanical loading. Studies have reported asymmetrical muscle activation, increased paraspinal muscle stiffness, and impaired neuromuscular control in individuals with AIS, all of which may limit the effectiveness of exercise-based interventions if not adequately addressed (3). Consequently, there is increasing interest in combining exercise therapy with adjunctive interventions that target these underlying biomechanical and muscular dysfunctions.

Manual therapy has emerged as a potential complementary approach in the conservative management of AIS. Techniques such as spinal mobilization, soft tissue manipulation, and myofascial release aim to improve joint mobility, reduce muscle tension, and enhance neuromuscular coordination. Recent randomized and pilot studies suggest that combining manual therapy with scoliosis-specific exercises may result in greater improvements in spinal curvature and functional outcomes compared to exercise alone (7, 8). However, current evidence remains limited, and findings are primarily derived from small-scale or methodologically heterogeneous studies.

Traditional Chinese manual therapy (TCM manipulation), including techniques such as Tuina, acupoint stimulation, and spinal mobilization, has been widely applied in musculoskeletal rehabilitation in China. This approach is based on the principles of restoring balance, promoting circulation, and regulating soft tissue function. Emerging clinical evidence indicates that TCM-based interventions may reduce pain, improve muscle symmetry, and positively influence functional outcomes in patients with scoliosis (8, 9). However, the proposed physiological mechanisms including effects on circulation, soft tissue extensibility, and neuromuscular responsiveness remain largely hypothetical and have not been rigorously validated in AIS populations (10).

Despite the growing clinical use of integrative rehabilitation approaches, high-quality evidence supporting the combined use of Traditional Chinese manual therapy and Schroth exercises remains limited. Existing studies are often constrained by small sample sizes, heterogeneous intervention protocols, and methodological limitations, which restrict the generalizability of their findings (5, 7, 8). Furthermore, few studies have systematically integrated radiographic, functional, and patient-reported outcomes to evaluate treatment effects comprehensively.

Therefore, there is a clear need for rigorously designed randomized controlled trials to evaluate the efficacy and safety of combining Traditional Chinese manual therapy with Schroth training in adolescents with AIS.

Accordingly, the aim of this study is to evaluate whether Traditional Chinese manual therapy combined with Schroth training is superior to Schroth training alone in improving spinal curvature, trunk symmetry, and health-related quality of life in adolescents with idiopathic scoliosis, while also assessing its safety and clinical feasibility as an integrative non-surgical treatment strategy.

## 2 Materials and methods

### 2.1 Study design

This study was conducted as a single-center, randomized, parallel-group controlled trial to evaluate the efficacy and safety of Traditional Chinese manual therapy combined with Schroth exercises in adolescents with idiopathic scoliosis. The trial followed a superiority design, with the intention of determining whether the combined intervention provided greater benefit than Schroth exercises alone. The methodological framework was based on standard recommendations for randomized controlled trial design and reporting. The study was reported in accordance with CONSORT (Consolidated Standards of Reporting Trials) guidelines. For each participant, the study timeline is defined relative to the date of randomization (designated as Week 0). The schedule of enrollment, interventions, and assessments is presented in Supplementary Table S1.

### 2.2 Study setting and study period

The study was carried out at Wenzhou People's Hospital. Recruitment and enrollment were conducted from January 2024 to December 2025. Participants were recruited through the outpatient spine clinic by trained clinical research staff who screened eligibility based on predefined inclusion and exclusion criteria. Each participant was followed for a total duration of 12 weeks, including a 2-week supervised intervention phase followed by a 10-week home-based rehabilitation period.

### 2.3 Participants

Adolescents diagnosed with idiopathic scoliosis were considered eligible if they had a Cobb angle between 10° and 45°, were between 8 and 18 years of age, were able to provide informed consent with guardian approval where required, and were capable of completing the treatment and follow-up procedures. AIS was defined according to standard clinical criteria, and the term “adolescents” was used to encompass a broad developmental range from early puberty to near skeletal maturity. Participants were excluded if they had severe scoliosis requiring surgical correction, congenital spinal deformity or other structural spinal disease, severe systemic illness, previous spinal surgery, or any condition that would interfere with participation or follow-up. Participants currently receiving brace treatment were excluded to avoid confounding effects of orthotic management on treatment outcomes. In addition, no participants received scoliosis bracing during the study period, and brace treatment was not permitted during the trial. Eligible participants were not restricted by curve pattern; therefore, thoracic, thoracolumbar, lumbar, and double major curves were included to reflect clinical heterogeneity. Curve type was recorded based on radiographic apex location for descriptive baseline characterization. Curve severity stratification (≤25° and >25°) was recorded descriptively. Formal skeletal maturity assessment (e.g., Risser or Sanders staging) and curve flexibility testing (e.g., bending radiographs) were not systematically available and therefore were not included in the study protocol. This represents a limitation of the study design and is acknowledged in the Limitations section. These eligibility criteria were broadly consistent with previous clinical studies investigating conservative interventions for AIS (3, 5, 6, 11).

### 2.4 Randomization and allocation concealment

Participants were randomly assigned in a 1:1 ratio to either the intervention or control group using stratified block randomization with variable block sizes of 4 and 6. Stratification was based on baseline Cobb angle severity (≤25° vs. >25°) to ensure balanced distribution between groups. The randomization sequence was generated by an independent statistician not involved in recruitment, treatment, or outcome assessment. Allocation concealment was ensured using sequentially numbered, opaque, sealed envelopes prepared by an independent research coordinator. After baseline assessment, the coordinator opened the next envelope in sequence to assign group allocation. Recruitment staff and therapists had no access to the allocation sequence prior to assignment, ensuring concealment and minimizing selection bias.

### 2.5 Blinding

Due to the nature of the intervention, blinding of participants and therapists was not feasible. However, outcome assessors and data analysts were fully blinded to group allocation. Specifically, radiographic, thermographic, and questionnaire data were collected and coded using anonymized participant identifiers. Two independent musculoskeletal radiologists performed Cobb angle measurements. Group allocation was not disclosed to assessors or statisticians until the completion of the primary analysis. Inter-rater and intra-rater reliability were assessed using intraclass correlation coefficients (ICC), demonstrating high measurement reliability. Unblinding was not permitted except in the case of safety concerns, in accordance with institutional ethical guidelines (9).

### 2.6 Interventions

Participants assigned to the control group received Schroth training alone. The Schroth program consisted of scoliosis-specific exercises tailored to the individual curve pattern and included three-dimensional postural correction, rotational angular breathing, postural stabilization, spinal elongation, and neuromuscular retraining. During the initial intensive phase, treatment was delivered under supervision twice daily, five days per week. After discharge, participants continued a structured home exercise program for 10 weeks, with follow-up support to encourage adherence (Supplementary Table S2). The use of Schroth exercises in AIS was supported by recent randomized trials and meta-analyses demonstrating improvements in spinal curvature, trunk rotation, and quality of life (3, 5, 6, 11, 12). Curve pattern classification was performed at baseline using Schroth-based scoliosis classification principles (thoracic, lumbar, and double-major curve patterns), and exercise prescriptions were individualized according to curve type and severity.

Participants allocated to the intervention group received Traditional Chinese manual therapy before each supervised Schroth session during the intensive phase. The manual treatment was delivered by trained practitioners and focused on relaxation of paraspinal soft tissues, targeted stimulation of relevant acupoints, and mobilization of spinal segments to improve flexibility and biomechanical balance. All manual therapy procedures were standardized according to a predefined treatment manual to ensure consistency across participants and therapists. All manual therapy procedures followed a standardized treatment protocol to ensure reproducibility across participants. The manual techniques included Tui Na soft tissue manipulation (kneading, rolling, and longitudinal stroking of paraspinal muscles from T1–L5), segmental spinal mobilization using graded pressure techniques, and myofascial release of tight musculature on the concave side of the curvature. Each manual therapy session lasted approximately 30 min, after which the participant performed the same Schroth training protocol used in the control group (Supplementary Table S3). Pressure intensity was standardized as mild-to-moderate, defined operationally as tissue mobilization sufficient to induce local muscle relaxation without pain or guarding response (rated ≤3 on a 10-point discomfort scale). Following the outpatient phase, participants in this group also continued the same 10-week home-based Schroth exercise program. Exercise progression criteria were predefined and included (1) ability to maintain corrected posture for at least 30 s, (2) correct execution of rotational angular breathing without compensatory trunk collapse, and (3) stable postural alignment during supervised assessment. Progression involved increasing hold duration, reducing external assistance, and advancing from assisted to independent corrective positioning.The rationale for this combined approach was that preparatory manual treatment might reduce soft tissue tension and improve segmental mobility, thereby enhancing the corrective effect of scoliosis-specific exercise (6–8). All interventions were delivered by certified physiotherapists trained in Schroth-based scoliosis-specific exercise therapy and licensed Traditional Chinese Medicine practitioners with at least 5 years of clinical experience in orthopedic manual therapy. All TCM practitioners held formal national certification in Traditional Chinese Medicine rehabilitation and had at least 5 years of clinical experience in musculoskeletal manual therapy. To ensure treatment standardization and reproducibility, both interventions were delivered according to a predefined standardized operating procedure (SOP), and therapists underwent training sessions prior to trial initiation. Training included a 2-week calibration program consisting of supervised practice sessions, protocol demonstrations, and inter-therapist agreement assessment to ensure consistency of technique delivery. Treatment fidelity was monitored through structured session checklists, periodic supervisor audits, and review of patient exercise logs. Adherence was defined as completion of ≥85% of scheduled supervised and home-based sessions. Total therapeutic exposure time was recorded for both groups, and Schroth exercise exposure was kept identical between groups to ensure that any between-group differences were attributable to the addition of manual therapy rather than differences in exercise dose.

### 2.7 Outcome measures

The primary outcome was the change in Cobb angle from baseline to week 12. Cobb angle was measured using standardized standing anteroposterior spinal radiographs at weeks 0 and 12 and remained the most widely accepted radiographic index for assessing scoliosis severity in clinical practice and research (4, 5). Radiographic measurements were independently performed by two blinded musculoskeletal radiologists, and inter- and intra-rater reliability were assessed using intraclass correlation coefficients (ICC). Radiographic imaging was performed following the ALARA (As Low As Reasonably Achievable) principle to minimize radiation exposure in adolescent participants. Standardized low-dose spinal radiographic protocols were used, and imaging was strictly limited to two time points (baseline and week 12) for research purposes. No additional radiographs were obtained beyond clinically required assessments.

Secondary outcomes included angle of trunk rotation, paraspinal thermal asymmetry, health-related quality of life, and safety. Angle of trunk rotation was measured at weeks 0, 2, and 12 using a scoliometer during the Adams forward-bending test. Paraspinal thermal asymmetry was evaluated at weeks 0, 2, and 12 by infrared thermography to identify temperature differences between the convex and concave sides of the trunk. Paraspinal thermal asymmetry (ΔT) was included as an exploratory physiological outcome reflecting potential differences in paraspinal soft tissue and autonomic balance; however, no established minimal clinically important difference (MCID) exists for AIS populations, and its clinical interpretation should therefore be considered exploratory. Health-related quality of life was assessed at weeks 0, 2, and 12 using the SRS-22r questionnaire, a validated scoliosis-specific patient-reported outcome measure. These measures were commonly used in contemporary AIS studies to capture structural, functional, and patient-centered treatment effects (3, 5, 6, 11, 12).

### 2.8 Safety assessment and adverse events

Safety assessment included systematic monitoring of adverse events and disease progression throughout the study period. Adverse events were defined as any untoward medical occurrence arising in a participant during the trial and were classified according to severity (mild, moderate, or severe) and their suspected relationship to the intervention. Serious adverse events (SAEs) were defined as events that were life-threatening, required or prolonged hospitalization, or resulted in significant disability or harm.

Adverse events were assessed at each study visit. Participants were specifically questioned regarding musculoskeletal discomfort, skin reactions, and neurological symptoms. All adverse events were documented, graded, and reported to the ethics committee in accordance with institutional requirements. Any serious adverse event prompted immediate review, and the trial could be modified or terminated if warranted by the safety findings. Participants who experienced trial-related adverse events were provided with appropriate emergency medical care by the investigators at no cost.

In addition to adverse event monitoring, disease progression was evaluated as an important safety parameter. Participants were regularly examined for clinical signs of worsening, including increased angle of trunk rotation (ATR) and observable deterioration in posture. If significant clinical progression was suspected, follow-up radiography was performed to confirm curve progression. When radiographic progression or clinically significant worsening was confirmed, the participant was withdrawn from the trial and referred for appropriate standard care.

### 2.9 Assessment schedule

Participants were assessed according to the predefined study schedule at baseline, after the 2-week intensive treatment phase, and at week 12 following completion of the home-based rehabilitation phase. This schedule allowed evaluation of both the immediate effects of the supervised intervention and the overall short-term efficacy of the complete rehabilitation program.

### 2.10 Data collection and monitoring

Designated outcome assessors, who were blinded to group assignment, recorded all measurements on standardized case report forms and subsequently entered the data into a secure electronic database for storage and analysis. The Academic Committee of Wenzhou People's Hospital functioned as the independent Data Monitoring Committee (DMC), overseeing trial conduct and participant safety. The DMC was independent of the sponsor and funder. In the event of safety concerns or unexpected serious adverse events, the DMC had the authority to recommend protocol modification or early trial termination. Data quality audits were conducted monthly by independent monitors. The statistician responsible for outcome analysis remained blinded to group allocation until completion of the primary analysis. To promote participant retention, reminder phone calls and follow-up messages were provided. For participants who discontinued or deviated from the intervention protocol, outcome data continued to be collected at all scheduled time points whenever possible. Participant adherence was monitored by reviewing treatment attendance, home exercise logs, and follow-up communication. During the home-based phase, participants recorded daily completion of prescribed exercises in standardized exercise logs, which were reviewed weekly by therapists via telephone follow-up or outpatient visits. Adherence was calculated as the proportion of completed prescribed sessions relative to the total planned sessions, and participants completing ≥85% of prescribed sessions were classified as adherent. All adverse events were documented and assessed according to their severity and possible relation to the intervention. Inter-rater and intra-rater reliability for Cobb angle measurements were evaluated using intraclass correlation coefficients (ICC), demonstrating high measurement consistency between raters.

### 2.11 Sample size

The sample size was determined *a priori* using G*Power software (version 3.1.9.7). Based on the primary outcome of change in Cobb angle, the required sample size was estimated from a previous randomized controlled trial (15). The calculation was conducted for a two-tailed independent-samples t-test with a significance level of 0.05 and a power of 80%. The analysis indicated that 72 participants (36 per group) were required. Assuming an attrition rate of approximately 20%, the final target sample size was increased to 86 participants, with 43 participants allocated to each group.

### 2.12 Statistical analysis

All statistical analyses were conducted using SPSS software. Continuous data were summarized as mean ± standard deviation, and categorical data were presented as frequencies and percentages, where appropriate. All analyses were performed according to the intention-to-treat principle, with a two-sided significance level of 0.05. The primary outcome, namely change in Cobb angle from baseline to week 12, was compared between groups using analysis of covariance (ANCOVA) adjusted for baseline Cobb angle. Model assumptions including normality (Shapiro–Wilk test), homogeneity of variance (Levene's test), and residual diagnostics were assessed to ensure validity of ANCOVA results. Results were expressed as adjusted mean differences with 95% confidence intervals, and effect sizes (partial eta-squared) were reported to quantify the magnitude of between-group differences. For secondary outcomes assessed at multiple time points, including angle of trunk rotation, paraspinal thermal asymmetry, and SRS-22r scores, linear mixed-effects models were applied to examine longitudinal changes. These models included fixed effects for group, time, and the group-by-time interaction, with participant treated as a random effect. The interaction term was used to assess whether outcome trajectories differed between groups over time.

To account for multiple comparisons across secondary outcomes, the false discovery rate (FDR) approach using the Benjamini–Hochberg procedure was applied. Secondary outcome analyses were considered exploratory and were interpreted cautiously.

Missing data were addressed under the missing-at-random assumption using mixed-model estimation. Multiple imputation was additionally performed using chained equations with 20 imputations, incorporating baseline demographic variables (age, sex), baseline clinical measures (Cobb angle, ATR, SRS-22r), and group allocation in the imputation model. Pooled estimates were derived using Rubin's rules to ensure robustness of findings.

## 3 Results

### 3.1 Participant flow and retention

**Figure 1:**
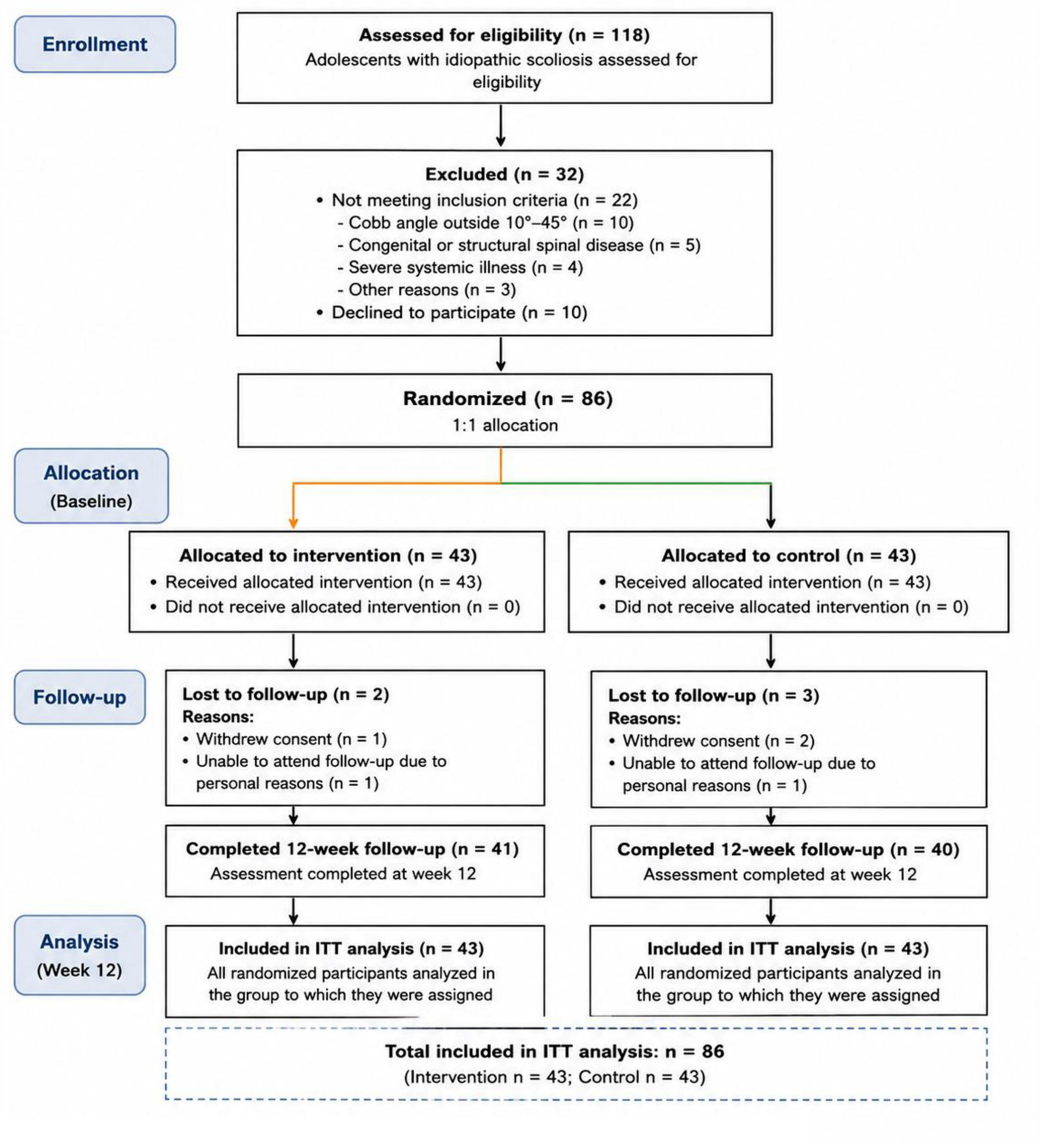
CONSORT flow diagram of participant enrollment and allocation. The diagram illustrates the flow of participants through each stage of the trial, including screening, eligibility assessment, randomization, allocation to intervention and control groups, follow-up, and analysis. Numbers of participants at each stage, as well as reasons for exclusion or dropout, are provided.

Between January 2024 and December 2025, 118 adolescents were screened for eligibility. After application of the predefined eligibility criteria, 86 participants were enrolled and randomly assigned to the intervention group (Traditional Chinese manual therapy combined with Schroth training; *n* = 43) or the control group (Schroth training alone; *n* = 43). During the 12-week study period, five participants discontinued the intervention, including two in the intervention group and three in the control group, mainly because of personal reasons or non-adherence. Week-12 outcome data were available for 81 participants. All randomized participants (*n* = 86) were included in the primary analysis according to the intention-to-treat principle. Missing outcome data were limited to 5 participants (5.8%) at week 12.The overall retention rate was 94.2%. Reasons for loss to follow-up were withdrawal of consent and inability to continue participation due to personal reasons, as detailed in the CONSORT flow diagram (Figure 1).

### 3.2 Baseline characteristics

The two groups were comparable at baseline. No statistically significant between-group differences were identified in age, sex distribution, baseline Cobb angle, angle of trunk rotation (ATR), paraspinal thermal asymmetry (ΔT), or SRS-22r score. In addition to demographic and clinical variables, baseline radiographic characteristics included curve type distribution (thoracic, thoracolumbar, lumbar, and double major curves), which showed no significant differences between groups (Table 1). Curve severity was also comparable between groups when stratified into mild (10°–25°) and moderate (26°–45°) categories. These findings indicate that randomization achieved an adequate balance in demographic and clinical characteristics before treatment initiation. Detailed baseline data are provided in Table 1.

**Table 1:** Baseline characteristics of participants.

Variable	Intervention (*n* = 43)	Control (*n* = 43)	*p*-value
Age (years)	13.6 ± 2.2	13.4 ± 2.1	0.65
Female, *n* (%)	31 (72.1%)	30 (69.8%)	0.81
Cobb angle (°)	25.0 ± 6.3	25.3 ± 6.1	0.78
ATR (°)	7.6 ± 2.2	7.4 ± 2.1	0.70
ΔT (°C)	0.71 ± 0.19	0.69 ± 0.18	0.62
SRS-22r score	3.55 ± 0.52	3.52 ± 0.50	0.74
Curve type—Thoracic, *n* (%)	18 (41.9%)	17 (39.5%)	0.88
Curve type—Thoracolumbar, *n* (%)	12 (27.9%)	13 (30.2%)	0.88
Curve type—Lumbar, *n* (%)	8 (18.6%)	7 (16.3%)	0.88
Curve type—Double major, *n* (%)	5 (11.6%)	6 (14.0%)	0.88
Curve severity—Mild (10°–25°), *n* (%)	24 (55.8%)	25 (58.1%)	0.92
Curve severity—Moderate (26°–45°), *n* (%)	19 (44.2%)	18 (41.9%)	0.92

Values are presented as mean ± standard deviation unless otherwise indicated. *P*-values compare baseline characteristics between the intervention and control groups. ATR, angle of trunk rotation; ΔT, paraspinal thermal asymmetry; SRS-22r, Scoliosis Research Society-22 revised questionnaire.

### 3.3 Primary outcome

**Figure 2:**
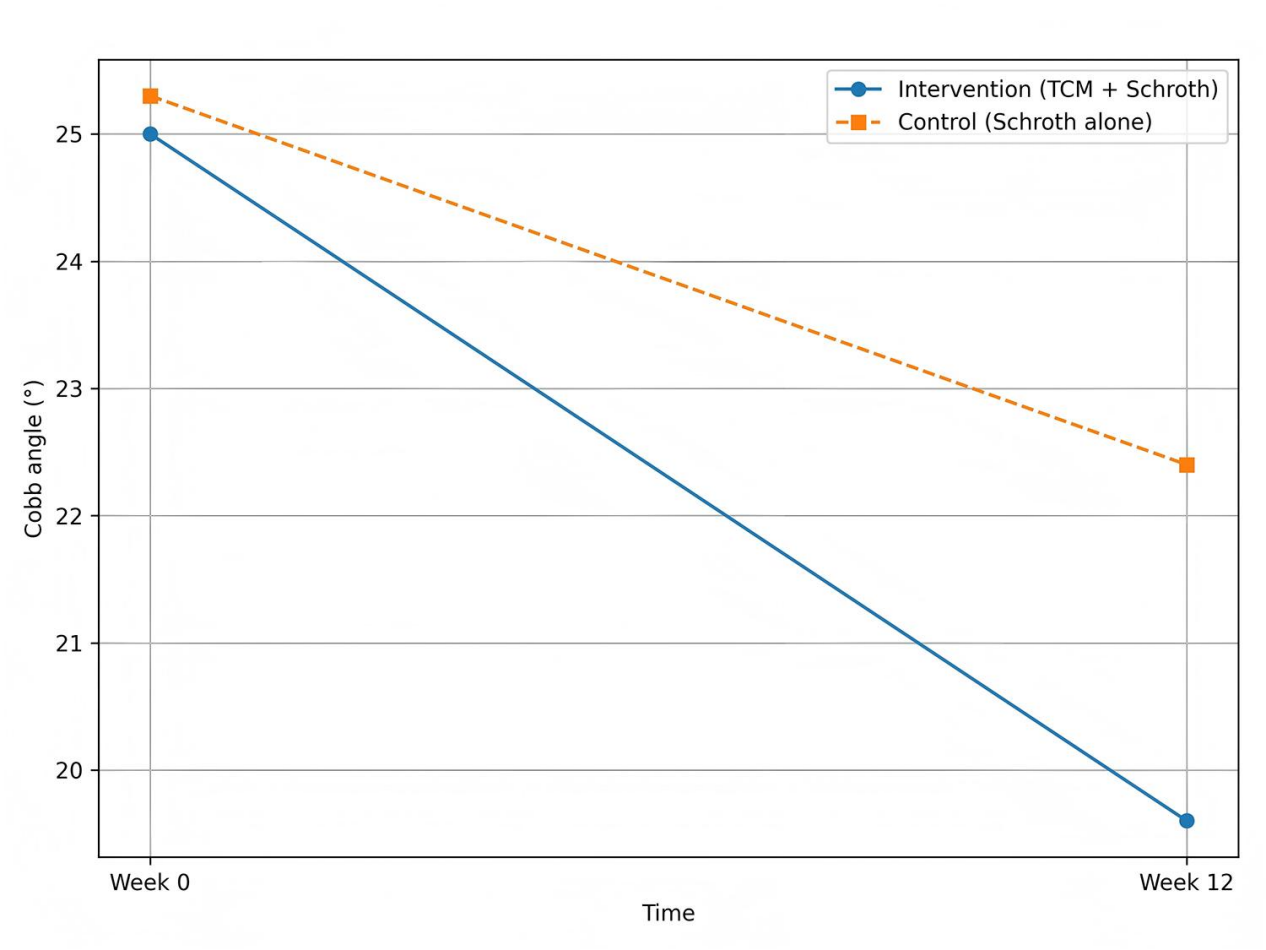
Change in cobb angle from baseline to week 12. Line graph showing the mean Cobb angle at baseline (Week 0) and at Week 12 for the intervention group (Traditional Chinese manual therapy combined with Schroth exercises) and the control group (Schroth exercises alone). Error bars represent standard deviation. The intervention group demonstrated a significantly greater reduction in Cobb angle compared to the control group.

The primary endpoint was change in Cobb angle from baseline to week 12. Both groups showed improvement over time, but the reduction was greater in the intervention group. After adjustment for baseline Cobb angle using analysis of covariance, the mean reduction was −5.4° (95% CI: −6.3 to −4.5) in the intervention group and −2.9° (95% CI: −3.7 to −2.1) in the control group. The adjusted between-group difference was −2.5° (95% CI: −3.8 to −1.3; *p* < 0.001), indicating a significant between-group difference favoring the intervention. A *post-hoc* responder analysis using a ± 5° threshold for clinically meaningful change was performed to further interpret individual-level responses. In the intervention group, 12 patients (27.9%) showed improvement (>5° reduction), 26 patients (60.5%) remained stable, and 5 patients (11.6%) showed worsening (>5° increase). In the control group, 6 patients (14.0%) showed improvement, 30 patients (69.8%) remained stable, and 7 patients (16.3%) showed worsening. Reviewer The change in Cobb angle across the study period is presented in Figure 2.

### 3.4 Secondary outcomes

Secondary outcomes were analyzed using linear mixed-effects models with fixed effects for group, time, and group-by-time interaction, and the resulting *p* values were adjusted using the false discovery rate method. Across all secondary endpoints, both groups improved over time, whereas the intervention group consistently showed greater improvement.

For angle of trunk rotation, the group-by-time interaction was significant (FDR-adjusted *p* < 0.01). By week 12, the mean reduction in ATR was −3.3° (95% CI: −3.9 to −2.7) in the intervention group, compared with −1.9° (95% CI: −2.4 to −1.4) in the control group.

For paraspinal thermal asymmetry, the intervention group also demonstrated a greater reduction than the control group, with a significant group-by-time interaction (FDR-adjusted *p* = 0.02). The mean decrease in ΔT at week 12 was −0.32 °C (95% CI: −0.38 to −0.26) in the intervention group and −0.18 °C (95% CI: −0.23 to −0.13) in the control group. As specified in the Methods, ΔT was an exploratory physiological outcome and should be interpreted cautiously without inference of direct clinical significance.

Health-related quality of life improved in both groups as reflected by SRS-22r scores, with superior gains in the intervention group. The group-by-time interaction was statistically significant (FDR-adjusted *p* < 0.01). At week 12, the mean increase in SRS-22r score was +0.66 (95% CI: 0.54 to 0.78) in the intervention group and +0.40 (95% CI: 0.30 to 0.50) in the control group.

**Figure 3:**
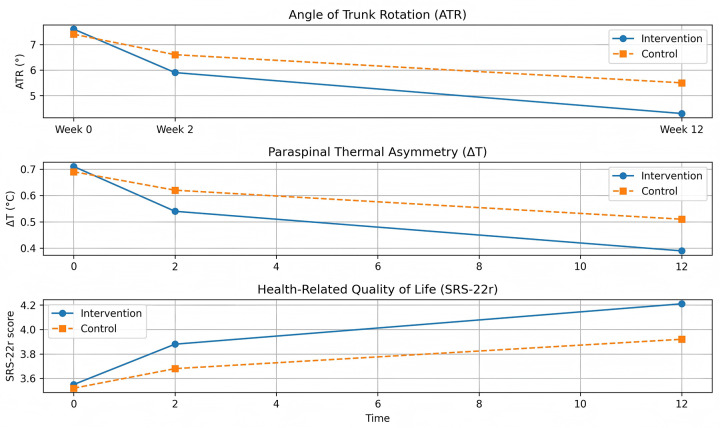
Longitudinal changes in secondary outcomes. Line graphs depicting the mean changes in **(A)** angle of trunk rotation (ATR), **(B)** paraspinal thermal asymmetry (ΔT), and **(C)** health-related quality of life (SRS-22r scores) over the study period (Weeks 0, 2, and 12) for the intervention and control groups. Error bars represent standard deviation. All outcomes showed significant group-by-time interactions, indicating greater improvements in the intervention group.

The detailed values for ATR, ΔT, and SRS-22r at baseline, week 2, and week 12 are presented in Table 2, while the corresponding longitudinal trends are illustrated in Figure 3.

**Table 2:** Secondary outcomes at baseline, week 2, and week 12.

Outcome	Time	Intervention	Control	Interaction *p*-value (FDR-adjusted)
ATR (°)	Week 0	7.6 ± 2.2	7.4 ± 2.1	
	Week 2	5.9 ± 1.9	6.6 ± 1.8	
	Week 12	4.3 ± 1.5	5.5 ± 1.6	<0.01
ΔT (°C)	Week 0	0.71 ± 0.19	0.69 ± 0.18	
	Week 2	0.54 ± 0.17	0.62 ± 0.16	
	Week 12	0.39 ± 0.14	0.51 ± 0.15	0.02
SRS-22r	Week 0	3.55 ± 0.52	3.52 ± 0.50	
	Week 2	3.88 ± 0.45	3.68 ± 0.47	
	Week 12	4.21 ± 0.34	3.92 ± 0.38	<0.01

Values are presented as mean ± standard deviation. Interaction *p*-values represent group × time effects derived from linear mixed-effects models and were adjusted for multiple comparisons using the false discovery rate (FDR) method. ATR, angle of trunk rotation; ΔT, paraspinal thermal asymmetry; SRS-22r, Scoliosis Research Society-22 revised questionnaire.

### 3.5 Safety outcomes

No serious adverse events were reported during the trial. Mild to moderate adverse events occurred in a small number of participants and included transient muscle soreness (*n* = 6), mild skin irritation (*n* = 3), and temporary fatigue (*n* = 4). All events resolved without hospitalization, additional intervention, or lasting harm. There was no significant difference between groups in the frequency of adverse events (*p* = 0.68). Disease progression was also monitored throughout follow-up, and no participant developed clinically significant deterioration requiring withdrawal from the study or referral for alternative treatment.

### 3.6 Adherence and feasibility

Adherence to the supervised treatment phase exceeded 90% in both groups. During the 10-week home-based phase, adherence remained acceptable, reaching 87% in the intervention group and 84% in the control group. Together with the low dropout rate and favorable safety profile, these findings support the feasibility and tolerability of the combined intervention in adolescents with idiopathic scoliosis.

### 3.7 Sensitivity analysis

Sensitivity analysis using multiple imputation for missing data yielded findings consistent with the primary intention-to-treat analysis. No meaningful differences were observed in the direction or significance of treatment effects, supporting the robustness of the main results.

## 4 Discussion

In this randomized controlled trial involving adolescents with idiopathic scoliosis (AIS), we found that adding Traditional Chinese manual therapy to a standardized Schroth exercise program resulted in significantly greater improvements in structural and functional outcomes compared with Schroth exercises alone. Specifically, the combined intervention produced larger reductions in Cobb angle and angle of trunk rotation (ATR), and greater changes in paraspinal thermal asymmetry (ΔT, interpreted as an exploratory physiological marker), as well as more substantial gains in health-related quality of life (SRS-22r) over 12 weeks. However, these findings should be interpreted as short-term statistical differences rather than definitive clinical improvements, particularly for radiographic outcomes.This study excluded patients receiving brace treatment; therefore, results should not be interpreted as evidence that physiotherapy alone can replace standard brace management in adolescents with moderate AIS. The 12-week follow-up period captures short-term treatment response but is insufficient to evaluate curve stability or progression, which typically requires longer follow-up intervals (≥6 months) in AIS. Therefore, the present findings should be interpreted as early radiographic changes rather than indicators of long-term stability. An early improvement pattern was observed in several outcomes, with a substantial proportion of the total change occurring within the first two weeks. This may reflect early neuromotor adaptation, learning effects, and rapid postural adjustment associated with scoliosis-specific exercise and manual therapy. However, the present study was not designed to assess the time course or plateau of response; therefore, it cannot determine whether early changes represent transient adaptation or sustained structural correction.

### 4.1 Principal findings and comparison with previous research

Our primary outcome of significantly greater Cobb angle reduction in the intervention group is consistent with evidence demonstrating the effectiveness of intensive physiotherapeutic scoliosis-specific exercise (PSSE) programs, including Schroth methods, for curve improvement in AIS populations (13–15). Recent meta-analyses have confirmed that Schroth exercises significantly reduce Cobb angle and ATR compared with conventional or no exercise (14, 16, 17). For instance, Schreiber et al. reported that Schroth exercise resulted in clinically meaningful improvements in spinal curvature relative to standard physiotherapy or observation (16), and Romano et al. demonstrated enhanced outcomes with individualized 3-D exercise regimens (17). Our findings extend this evidence by suggesting that the addition of Traditional Chinese manual therapy may be associated with additional short-term improvement, although confirmation in larger multicenter trials with longer follow-up is required. In the present study, the observed between-group difference in Cobb angle was relatively small. Considering the known measurement variability of Cobb angle (approximately 3°–5°), this difference should be interpreted cautiously and may fall within the range of radiographic measurement error. Therefore, while statistically significant, the clinical relevance of this change remains uncertain, particularly in mild AIS cases. The greater reduction in ATR observed in the combined group aligns with prior evidence that targeted three-dimensional corrective exercises improve rotational deformity in AIS (13–16). ATR measures reflect rotational components of spinal deformity that contribute to cosmetic and functional impairment (6, 18). The observed differences suggest that manual therapy may enhance soft tissue flexibility and neuromuscular coordination, potentially facilitating more effective execution of corrective exercises. However, the durability of these changes over time cannot be determined from the present 12-week study. Taken together, these findings suggest a potential synergistic effect between manual therapy and PSSE, although this requires further confirmation in larger multicenter trials. Our results support the notion that augmenting Schroth PSSE with manual therapy may further enhance neuromuscular coordination and soft tissue flexibility, leading to more pronounced rotational corrections, as suggested in emerging clinical series (19, 20).

Our study extends these findings by suggesting that the addition of Traditional Chinese manual therapy may provide additional benefit beyond exercise alone, although confirmatory multicenter studies are required. Although the literature on thermographic outcomes in AIS is limited, this observation parallels reports that PSSE and manual interventions improve muscular symmetry and segmental balance (20, 21). Therefore, ΔT should be considered a descriptive physiological marker rather than a validated clinical endpoint.

Quality of life, assessed by the SRS-22r measure, improved significantly in both groups, with greater gains in the intervention group. This finding aligns with prior research demonstrating the psychosocial and physical benefits of Schroth-based PSSE on patient-reported outcomes (22, 23). Improvements in self-image and function have been consistently reported with scoliosis-specific rehabilitation (22, 24). Our findings underscore the importance of integrating patient-centered outcomes with objective measures in evaluating conservative AIS interventions, as structural improvements alone may not fully capture the impact on health-related quality of life.

Safety outcomes indicated that both interventions were well tolerated, with only mild and transient adverse events. This is consistent with previous reports supporting the safety of conservative scoliosis management (25, 26). However, the short follow-up period limits interpretation of long-term safety and sustained tolerability.

### 4.2 Mechanistic considerations

The observed short-term differences may reflect combined effects of manual therapy and exercise on movement performance and rehabilitation engagement. Schroth exercises target active postural correction and motor control (27), while manual therapy may influence tissue extensibility and joint mobility (28–30). These potential explanations remain hypothetical and were not directly examined in this study. In particular, neuromuscular coordination, proprioceptive adaptation, and autonomic regulation were not measured and therefore cannot be inferred from the present data. Accordingly, mechanistic interpretations should be considered hypothesis-generating only.

### 4.3 Clinical and research implications

Our findings have several implications for clinical practice and future research. First, they support the inclusion of manual therapy as a complementary modality within comprehensive conservative management of AIS. While Schroth and other PSSE programs are recommended in clinical guidelines (17), evidence for adjunctive manual techniques remains limited. Our results suggest that adding manual therapy may enhance the effectiveness of standard exercise protocols, potentially supporting more efficient improvements in spinal curvature and trunk symmetry. Second, the improvements in patient-reported outcomes highlight the importance of using validated measures such as SRS-22r alongside objective structural metrics. Incorporating both types of outcomes allows clinicians to more accurately evaluate treatment efficacy and tailor interventions to the specific functional and psychosocial needs of adolescents with AIS. Third, the high adherence and low dropout rates observed indicate that multimodal conservative treatments are feasible and acceptable in adolescent populations. This suggests that adherence-promoting strategies, such as structured supervision and follow-up communication, are essential for maintaining engagement during home-based rehabilitation phases, which may enhance long-term treatment effectiveness. Finally, our findings support the expansion of future randomized controlled trials that examine long-term outcomes, include multicenter designs, and compare combined interventions with other conservative strategies such as bracing or innovative PSSE modalities (31, 32). Extended observation periods would be particularly valuable for assessing the durability of structural, functional, and patient-centered improvements, and could inform guidelines for optimal intervention timing and intensity.

### 4.4 Limitations

Despite these strengths, several limitations merit consideration. First, although this study employed rigorous randomization and blinding of assessors, participants and therapists were not blinded due to the nature of the interventions, which may introduce performance bias. Second, the relatively short follow-up period (12 weeks) limits conclusions about the long-term sustainability of observed changes. The 12-week radiographic assessment captures early treatment response but is insufficient to evaluate curve stability or progression, which typically requires longer follow-up intervals (≥6 months) in AIS. Third, although ΔT was included as an exploratory outcome, its clinical and mechanistic relevance remains unvalidated and no minimal clinically important difference exists. Fourth, the single-center design may limit generalizability to broader clinical and healthcare settings. In particular, potential cultural and practice-specific aspects of Traditional Chinese manual therapy may limit external validity across different healthcare systems. Fifth, the inclusion of participants aged 8–18 years introduces potential heterogeneity in skeletal maturity. Although the study population was defined as adolescents within this age range, this includes variability in skeletal maturity, and some participants may have been pre-mature or near skeletal maturity. Because skeletal maturity assessment (e.g., Risser or Sanders staging) was not systematically recorded, residual heterogeneity in growth status and curve progression risk cannot be excluded. Sixth, although statistically significant, the observed differences in Cobb angle were small and within the range of known measurement error (approximately 3°–5°), limiting their clinical interpretability. Seventh, thermal asymmetry, while informative for soft tissue balance, remains an indirect measure and its clinical significance requires further validation (33). Eighth, according to current clinical guidelines, bracing is recommended for moderate AIS (Cobb angle 25°–45°) in skeletally immature patients; therefore, the present study applies only to non-braced participants and the results should not be generalized to standard brace-treated populations. The study included an intermediate assessment at 2 weeks; however, the design was not powered to analyze trajectory or time-to-maximum correction. Therefore, the observed early improvements should not be interpreted as evidence of long-term stabilization or maximal treatment effect. Future studies could expand on these aspects by including longer follow-up durations, multiple centers, and complementary imaging or biomechanical assessments to corroborate findings.

### 4.5 Strengths

This trial also has notable strengths, including a well-defined intervention protocol, use of standardized outcome measures recommended in scoliosis research (34), and comprehensive safety monitoring. The integration of both objective and patient-reported outcomes enhances the clinical relevance and interpretability of the findings, providing a robust framework for assessing the efficacy of combined conservative interventions in AIS.

## 5 Conclusion

In adolescents with idiopathic scoliosis, the addition of Traditional Chinese manual therapy to a Schroth exercise regimen was associated with short-term improvements in spinal curvature (Cobb angle), trunk rotation (ATR), paraspinal symmetry (ΔT), and health-related quality of life (SRS-22r) compared with Schroth exercises alone. Both interventions were safe and well tolerated. However, the observed radiographic differences were small and should be interpreted cautiously given known measurement variability and the short follow-up period. These findings do not demonstrate clinically meaningful structural correction, long-term effectiveness, or superiority over standard bracing-based management in indicated patients. Therefore, this intervention should be considered a short-term adjunct to conservative rehabilitation rather than a standalone treatment for adolescent idiopathic scoliosis.

## Data Availability

The original contributions presented in the study are included in the article/Supplementary Material, further inquiries can be directed to the corresponding author.
