# Supplementary material for: Integrative conservative rehabilitation for adolescent idiopathic scoliosis: a randomized controlled trial of traditional Chinese manual therapy combined with Schroth training
Source: Front Pediatr. 2026 Jul 30;14:1868538. doi: 10.3389/fped.2026.1868538 (PMC13469262; doi:10.3389/fped.2026.1868538)
Supplement: Supplementary file 1 [file Table1.docx]

**Table S1.** **Schedule of enrollment, interventions, and assessments**

| **Study period** | **Screening** | **Baseline**  **(Week 0)** | **Week 2** | **Week 12** |
| --- | --- | --- | --- | --- |
| Eligibility screening | ✓ |  |  |  |
| Informed consent | ✓ |  |  |  |
| Randomization |  | ✓ |  |  |
| Schroth training (control) |  | ✓ | ✓ | ✓ |
| Manual therapy + Schroth (intervention) |  | ✓ | ✓ | ✓ |
| Cobb angle (radiograph) |  | ✓ |  | ✓ |
| ATR (scoliometer) |  | ✓ | ✓ | ✓ |
| Paraspinal ΔT (thermography) |  | ✓ | ✓ | ✓ |
| SRS-22r questionnaire |  | ✓ | ✓ | ✓ |
| Adverse events monitoring |  | ✓ | ✓ | ✓ |

Check marks (✓) indicate the time points at which study procedures, interventions, or assessments were performed. ATR, angle of trunk rotation; ΔT, paraspinal thermal asymmetry; SRS-22r, Scoliosis Research Society-22 revised questionnai
