# Supplementary material for: Integrative conservative rehabilitation for adolescent idiopathic scoliosis: a randomized controlled trial of traditional Chinese manual therapy combined with Schroth training
Source: Front Pediatr. 2026 Jul 30;14:1868538. doi: 10.3389/fped.2026.1868538 (PMC13469262; doi:10.3389/fped.2026.1868538)
Supplement: Supplementary file 2 [file Table2.docx]

**Table S2. Schroth Therapy**

| **Step** | **Exercise Component** | **Procedure Description** |
| --- | --- | --- |
| 1 | Postural Awareness Training | Patient is guided to recognize their scoliosis curve pattern using mirrors and verbal cues. Education is provided on three-dimensional scoliosis correction principles (derotation, deflexion, elongation). |
| 2 | Corrective Breathing (Rotational Angular Breathing, RAB) | Patient performs segmental breathing into the concave side of the ribcage while maintaining corrective posture, emphasizing expansion of collapsed areas and active exhalation to promote rib derotation. |
| 3 | Pelvic Alignment and Stabilization | Exercises to correct pelvic tilt and rotation are performed in standing, sitting, or supine positions, using isometric activation of gluteal and abdominal muscles to stabilize the pelvis. |
| 4 | Spinal Elongation and Axial Stretching | Patient actively lengthens the spine against gravity (in seated or standing positions), with manual or elastic band assistance to increase vertebral spacing and reduce curve compression. |
| 5 | Asymmetric Strengthening | Targeted strengthening of trunk muscles on the convex side to support curve correction, using resistance bands or body weight. |
| 6 | Symmetric Integration Exercises | Functional activities performed in corrected posture (e.g., squatting, stepping, reaching), reinforcing muscle memory of the corrected alignment. |
| 7 | Relaxation and Recovery | Gentle stretching and breathing exercises to relax overactive musculature and prevent fatigue after corrective exercises. |

RAB, rotational angular breathing. The Schroth therapy program was individualized according to each participant’s curve pattern and functional status.
