# Supplementary material for: Integrative conservative rehabilitation for adolescent idiopathic scoliosis: a randomized controlled trial of traditional Chinese manual therapy combined with Schroth training
Source: Front Pediatr. 2026 Jul 30;14:1868538. doi: 10.3389/fped.2026.1868538 (PMC13469262; doi:10.3389/fped.2026.1868538)
Supplement: Supplementary file 3 [file Table3.docx]

**Table S3. TCM Manipulation**

| **Step** | **Manipulation Technique** | **Procedure Description** |
| --- | --- | --- |
| 1 | Gunfa (Rolling) and Zhang Roufa (Palm Kneading) | Patient in prone position. Apply rolling and palm kneading to the muscles along the Bladder Meridian of the back and the Jiaji region, moving back and forth several times. Palpate for jingbi (tender/obstructed) points. Use Roubo Fa (kneading and plucking) to relax tense muscles; use rolling and palm kneading on soft or weak musculature. Duration: ~10 min. |
| 2 | Yizhichan Tuifa (One-finger Zen Pushing) with Zhi Roufa (Finger Kneading) | Based on syndrome differentiation, select acupoints such as Feishu (BL13), Xinshu (BL15), Ganshu (BL18), Pishu (BL20), Weishu (BL21), Shenshu (BL23), Dachangshu (BL25). Push along the point using the one-finger Zen technique, pausing at each point for finger kneading 1–2 min, until a tolerable aching/distending sensation occurs. Duration: ~5 min. |
| 3 | Zhang Ca Fa (Palm Rubbing) and Niejifa (Spinal Pinching) | Apply a small amount of Vaseline or similar medium along the Du Mai (Governor Vessel), Jiaji, and Bladder Meridian areas. Perform palm rubbing in straight, back-and-forth movements until the skin is slightly red and warm, then pinch along the spine for 3–5 passes. |
| 4 | Rhythmic Spinal Oscillation | Place one palm over the cervicothoracic junction or a selected spinal segment for stabilization, and the other palm on the lumbosacral midline. Using the spine as the axis, both palms work together to rhythmically oscillate the spine and pelvis, gradually increasing amplitude within patient tolerance. |
| 5 | Seated Rotational and Extension Stretch | Patient seated, arms crossed hugging a pillow, with lower limbs and pelvis stabilized. Practitioner stands behind and slightly to the side, placing one hand on the convex side of the curve for support and the other under the axilla to grasp the opposite shoulder. Guide the patient into slight forward flexion, then rotation and extension toward the same side. Repeat for the opposite side. |
| 6 | Bilateral Axillary Lift and Thoracic Distraction | Practitioner passes both arms under the patient’s axillae, grasping the upper arms, and gently lifts upward and backward to distract and stretch the thoracic spine, producing mild joint loosening and widening of intervertebral spaces. |
| 7 | Finishing Technique | Conclude with gentle tapping or kneading to relax tissues. Total treatment duration: approximately 30–40 min per session. |

BL denotes Bladder Meridian acupoints. TCM manipulation was administered by trained practitioners according to the standardized study protocol, with a total treatment duration of approximately 30–40 min per session.
